# Supplementary material for: HIVprotI: an integrated web based platform for prediction and design of HIV proteins inhibitors
Source: J Cheminform. 2018 Mar 9;10:12. doi: 10.1186/s13321-018-0266-y (PMC5845081; doi:10.1186/s13321-018-0266-y)
Supplement: Supplementary file 2 — Additional file 2. Source code of HIVProtI web server. [file 13321_2018_266_MOESM2_ESM.zip › HIVprotI_Source-code/hivprotI/weka-3-6-6/doc/weka/classifiers/functions/supportVector/NormalizedPolyKernel.html]

NormalizedPolyKernel


---


|  |  |  |
| --- | --- | --- |
| **Overview** \| **Package** \| **Class** \| **Tree** \| **Deprecated** \| **Index** \| **Help** \| **Weka's home** | |  |
| **PREV CLASS**   **NEXT CLASS** | **FRAMES**    **NO FRAMES**     **All Classes** |  |
| SUMMARY: NESTED \| FIELD \| CONSTR \| METHOD | DETAIL: FIELD \| CONSTR \| METHOD |  |


---


## weka.classifiers.functions.supportVector Class NormalizedPolyKernel

```
java.lang.Object
  weka.classifiers.functions.supportVector.Kernel
      weka.classifiers.functions.supportVector.CachedKernel
          weka.classifiers.functions.supportVector.PolyKernel
              weka.classifiers.functions.supportVector.NormalizedPolyKernel
```

**All Implemented Interfaces:**: java.io.Serializable, CapabilitiesHandler, OptionHandler, RevisionHandler

---

``` public class NormalizedPolyKernel extends PolyKernel ```

The normalized polynomial kernel.
